# Inhibition of Aspergillus oryzae Mycelium Growth and Conidium Production by Irradiation with Light at Different Wavelengths and Intensities

**DOI:** 10.1128/spectrum.00213-21

**Published:** 2021-08-04

**Authors:** Shangfei Lin, Haokuan Qin, Xiaolin Zhang, Wenqi Li, Muqing Liu

**Affiliations:** a Institute of Future Lighting, Academy for Engineering and Technology, Fudan University, Shanghai, China; b Jihua Laboratory, Foshan City, Guangdong Province, China; c Department of Light Sources and Illuminating Engineering, School of Information Science and Technology, Fudan University, Shanghai, China; Universidade de Sao Paulo

**Keywords:** photoreaction, conidium, *Aspergillus oryzae*, light wavelength, light intensity

## Abstract

Aspergillus oryzae is a safe filamentous fungus widely used in the food, medicine, and feed industries, but there is currently not enough research on the light response of A. oryzae. In this study, 12 different light conditions were set and A. oryzae GDMCC 3.31 was continuously irradiated for 72 h to investigate the effect of light on mycelial growth and conidium production. Specifically, each light condition was the combination of one light wavelength (475, 520, or 630 nm) and one light intensity (20, 40, 60, or 80 μmol photon m^−2^ s^−1^). The results show that mycelium growth was inhibited significantly by green light (wavelength of 520 nm and intensities of 20 and 60 μmol photon m^−2^ s^−1^) and blue light (wavelength of 475 nm and intensity of 80 μmol photon m^−2^ s^−1^). The production of conidia was suppressed only by blue light (wavelength of 475 nm and intensities of 40, 60, and 80 μmol photon m^−2^ s^−1^), and those levels of inhibition increased when the intensity of blue light increased. When the strain was irradiated by blue light (80 μmol photon m^−2^ s^−1^), the number of conidia was 57.4% less than that of the darkness group. However, within our set range of light intensities, A. oryzae GDMCC 3.31 was insensitive to red light (wavelength of 630 nm) in terms of mycelium growth and conidium production. Moreover, interaction effects between light wavelength and intensity were found to exist in terms of colony diameter and the number of conidia. This research investigated the light response of A. oryzae, which may provide a new method to regulate mixed strains in fermented foods by light.

**IMPORTANCE** Studies on the monochromatic light response of Aspergillus nidulans and Neurospora crassa have gone deep into the molecular mechanism. However, research methods for the light response of A. oryzae remain in the use of white light sources. In this study, we first demonstrated that A. oryzae GDMCC 3.31 was sensitive to light wavelength and intensity. We have observed that blue light inhibited its growth and sporulation and the inhibitory effect increased with intensity. This research not only adds new content to the study of the photoreaction of Aspergillus but also brings new possibilities for the use of light to regulate mixed strains and ultimately improve the flavor quality of fermented foods.

## INTRODUCTION

Some species in the genus Aspergillus are widely used in the industrial production of fermented foods, industrial enzyme preparations, and secondary metabolites, such as Aspergillus niger and Aspergillus oryzae. As an environmental factor, light also affects Aspergillus (1–7). However, at present, research on the light response of fungi mainly focuses on the model species: for example, Aspergillus nidulans and Neurospora crassa (8–11). Many studies have shown that A. nidulans has various photoreceptors (12, 13), and its growth, spore formation, primary metabolism, and secondary metabolites are all regulated by light stimulation (5, 6, 10, 14–16). Aspergillus oryzae is a recognized safe-to-use species in the food industry and one of the strains used in the production of soybean paste and soy sauce, which is a traditional Chinese food seasoning. The growth, vegetative reproduction, and secondary metabolite yield of A. oryzae strains affect the production cycle and product quality of the food fermentation industry and enzyme industry. Therefore, research on the light response of A. oryzae is very necessary.

Fortunately, some researchers have conducted preliminary studies on the light response of the growth and reproduction of A. oryzae. It was first found in 2007 that in RIB40, a standard strain of Aspergillus oryzae, conidiation was repressed by white light at an intensity of 94.2 μmol photon m^−2^ s^−1^ and red light at an intensity of 75.5 μmol photon m^−2^ s^−1^, while one industrially applied strain showed no conserved result (17). Near-UV light at an intensity of 6 W/m^2^ was discovered to strongly inhibit the growth of germinated pellets of A. oryzae RIB40, and this effect could be weakened by TiO_2_ particles (0.05 g/liter) (18). A. oryzae F6, which was obtained after mutagenesis by UV light, formed ring-shaped colonies under the condition of 12 h of light/12 h of dark. In addition, its number of conidia and acid protease were also repressed by continuous white light at an intensity of 25 μmol photon m^−2^ s^−1^ (19). Ten laboratory A. oryzae strains were placed individually under a fluorescent lamp at a light intensity of 25 μmol photon m^−2^ s^−1^. After illumination, the growth rates of hyphae and conidia were compared with those in the dark group, and the results showed an inconsistent trend. Among them, A. oryzae RIB1187 responded positively to incubation under the light condition and produced more conidia than in the dark. However, A. oryzae RIB40 showed a negative light response (20). Furthermore, transcriptome analysis was conducted for these two strains, and 453 differentially expressed genes (DEGs) were identified. Among these 453 DEGs, 67 DEGs were jointly identified as related to light response (21). All of these results of mycelium growth and conidiation implied that there was an impact from light irradiation on A. oryzae and suggested that the effect of light irradiation on the growth and conidiation varies among A. oryzae strains. However, the factors associated with light irradiation, including light quality (wavelength), intensity, irradiation time, and irradiation dosage, were not taken into consideration comprehensively. Light sources with a wide spectrum, such as fluorescent lamps, are not the most suitable light source for studying the light response of A. oryzae, because the photoreceptors of fungi are generally sensitive to monochromatic light at a specific wavelength (6, 13, 16, 22, 23). Monochromatic light sources, such as light-emitting diodes and lasers, are perhaps a better choice.

Here, we used light wavelength and light intensity as two impact factors and set up 12 different light conditions to investigate how Aspergillus oryzae GDMCC 3.31 responds to different light irradiation parameters in terms of growth of hyphae and conidia. Furthermore, we used two-way analysis of variance (ANOVA) to analyze the effect of interaction between light wavelength and intensity on these responses. This lays the seeds for future research to find out the photoreceptor and verify its photoresponse range.

## RESULTS

### Effects of light irradiation on mycelial growth.

**FIG 1:**
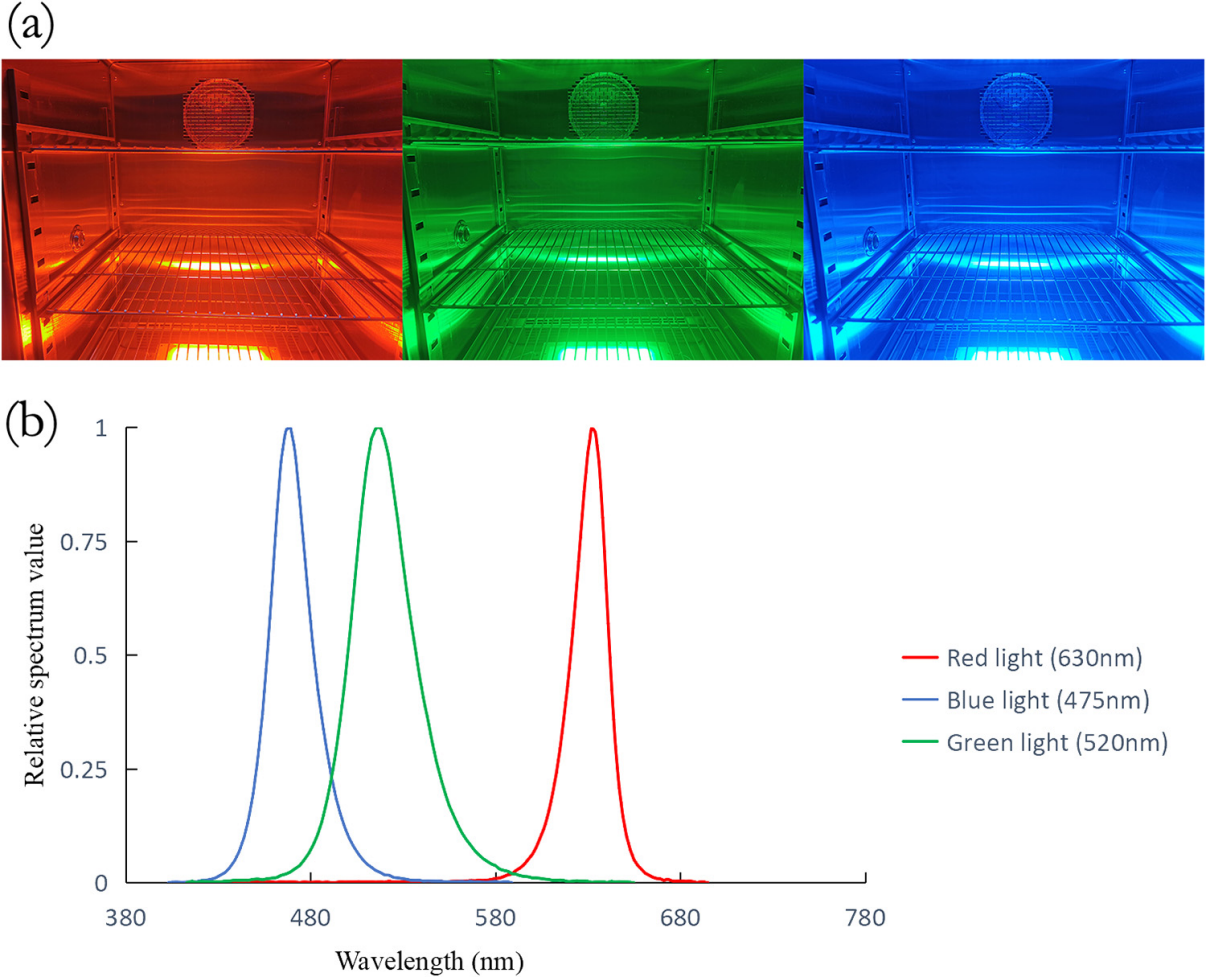
Illumination incubator and the spectrum of LED light sources. (a) Illumination incubator. (b) The spectrum of light sources used in this study, including blue light (475 nm), green light (520 nm), and red light (630 nm).

**FIG 2:**
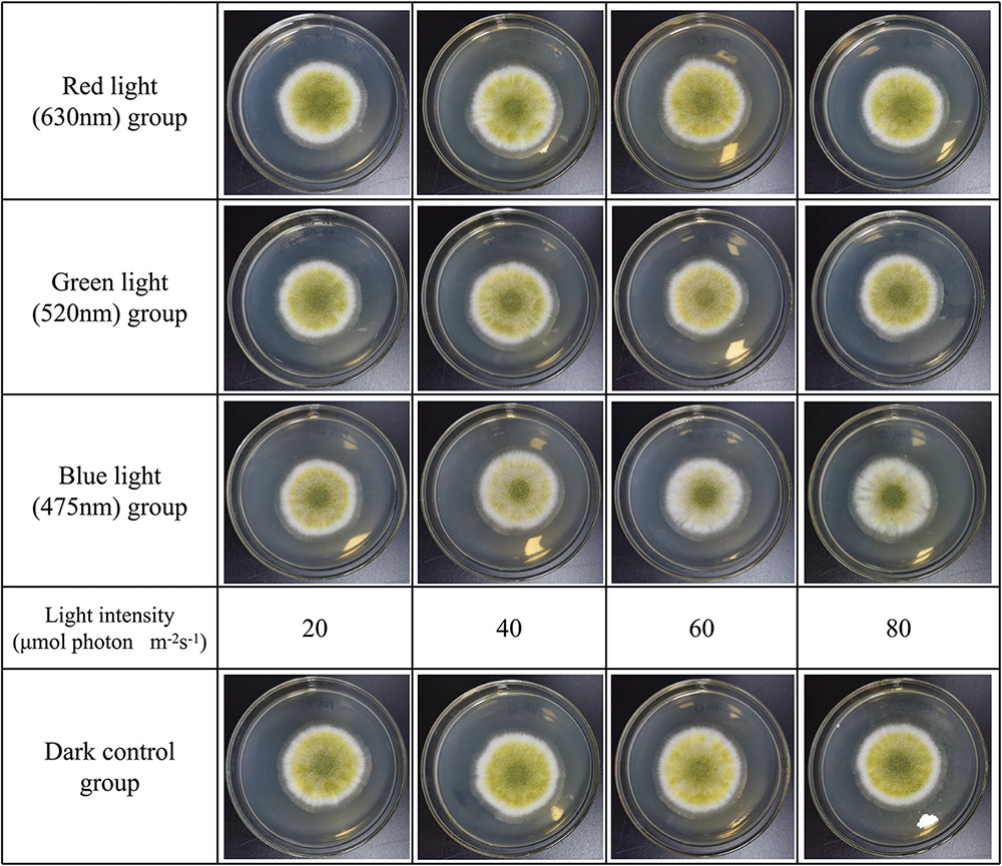
Influence of different types of light irradiation on A. oryzae GDMCC 3.31. Strains were incubated on PDA plates for 72 h at 30°C. Until incubation, each plate was examined and photos were obtained.

To investigate if mycelial growth was affected by light, the effect of light wavelength on mycelial growth was first observed. The light irradiation parameters used are shown in Table 1. The illumination incubator and spectrum of LED light sources used are shown in Fig. 1. The plate was point inoculated with 10 μl of inoculum solution, and A. oryzae was incubated for 3 days. Until 72 h, obvious colonies formed in the plate. The influence of different types of light irradiation on the mycelial growth of A. oryzae was examined (Fig. 2). The mycelium in each group developed into a round colony. The white hyphae and yellow-green mature spores indicated that all strains were developing normally. However, groups under blue light irradiation not only had a smaller range of white hyphae, but also had a smaller yellow-green range than that of the group in the dark.

**TABLE 1:** Light irradiation parameters

Irradiation parameter	Result under^*a*^:											
	Blue light				Green light				Red light			
Light wavelength (nm)	475				520				630			
Light intensity (μmol photon m^−2^ s^−1^)	20	40	60	80	20	40	60	80	20	40	60	80
Irradiance (W m^−2^)	4.59 ± 0.15	9.36 ± 0.09	14.70 ± 0.00	19.87 ± 0.17	4.94 ± 0.38	8.83 ± 0.07	13.27 ± 0.05	17.87 ± 0.05	3.94 ± 0.38	7.67 ± 0.03	11.97 ± 0.05	15.63 ± 0.09

a Irradiance values are expressed as means ± standard deviations.

### Effects of different light intensities on mycelial growth under the same light wavelength.

**FIG 3:**
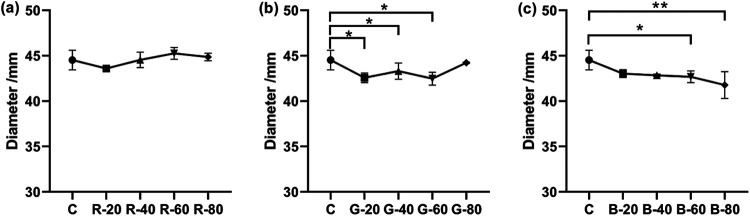
Colony diameters of groups under different light intensities. (a) Colony diameters of the red light group with different intensities. (b) Colony diameters of the green light group. (c) Colony diameters of the blue light group. In panel a, the points on the *x* axis from left to right represent the dark control group and the red light groups, with intensities of 20, 40, 60, and 80 μmol photon m^−2^ s^−1^. Panels b and c are marked in the same way. The error bars represent the standard deviation. Significance: *, *P* < 0.05; **, *P* < 0.01.

When the light wavelength was maintained, the effect of light intensity on mycelial growth was investigated. All results were recorded in Table 2. As shown in Fig. 3a, regardless of the intensity, the average diameter of each group under red light irradiation (630 nm) was near that of the dark group. When the groups were under green light irradiation (520 nm), although the mycelial growth was slightly suppressed by the low, middle, and relatively high light intensities (*P* = 0.0188, 0.0320, and 0.0122, respectively), the colony diameter of the green light group (80 μmol photon m^−2^ s^−1^) was found to have no significant difference from the darkness group. When the strain was irradiated by blue light (475 nm), the mycelial growth showed different results in terms of light intensities. The blue light with intensities of 20 and 40 μmol photon m^−2^ s^−1^ resulted in slightly smaller colony diameters than the darkness group, but without significant difference. Although there seemed to be a slow downward trend in mycelial growth when the blue light intensities increased, only intensities of 60 and 80 μmol photon m^−2^ s^−1^ had significant inhibition, which slowed down growth by 4.1% and 6.2%, respectively. In terms of mycelial growth, A. oryzae GDMCC 3.31 showed selectivity in the light wavelength. Within the set range of intensities, A. oryzae GDMCC 3.31 was not sensitive to the red light, while the green light and the blue light had general suppression. Besides, under the specific wavelength condition, the strain’s mycelial growth under light irradiation was also affected by the light intensity.

**TABLE 2:** Colony diameter results under different light wavelengths and intensities

Light intensity (μmol photon m^−2^ s^−1^)	Colony diam (mm) under^*a*^:			
	Darkness	Red light	Green light	Blue light
20	44.52 ± 1.09^ABCDE^	43.59 ± 025	42.57 ± 0.52^A^	43.03 ± 0.43
40		44.55 ± 0.85	43.30 ± 0.89^B^	42.85 ± 0.13
60		45.26 ± 0.65^FG^	42.47 ± 0.71^CF^	42.68 ± 0.64^DG^
80		44.86 ± 0.42^H^	44.22 ± 0.08^I^	41.77 ± 1.48^EHI^

a The values are expressed as means ± standard deviations. Each treatment was repeated at least 3 times. Different letters in columns and rows represent statistically different mean values (*P* < 0.05).

### Effects of different light wavelengths on mycelial growth under the same light intensity.

**FIG 4:**
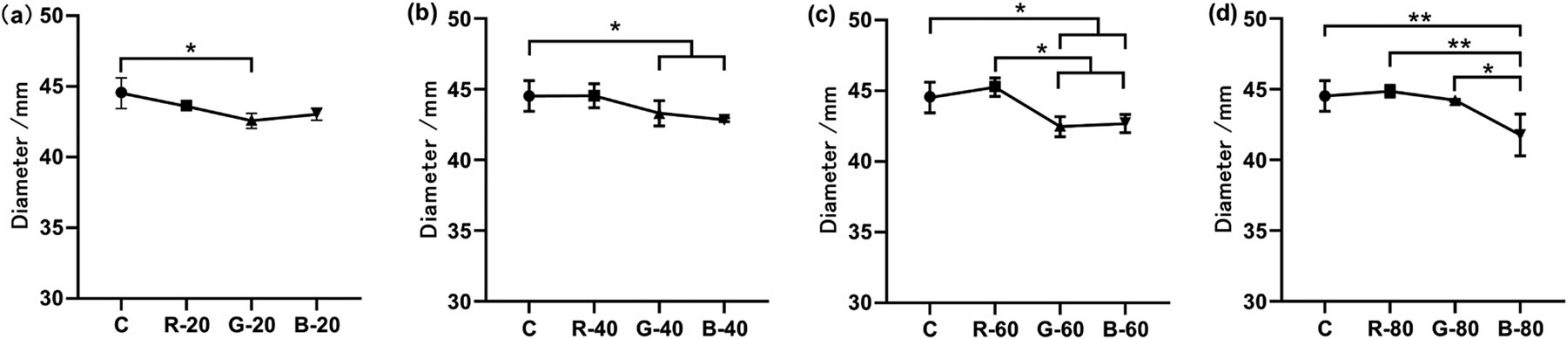
Colony diameters of groups under different light wavelengths. Panel a shows the colony diameters of the groups with different light wavelengths under the same intensity (20 μmol photon m^−2^ s^−1^). Panels b, c, and d are marked in the same way with the respective intensities of 40, 60, and 80 μmol photon m^−2^ s^−1^. The error bars represent the standard deviation. Significance: *, *P* < 0.05; **, *P* < 0.01.

When the light conditions were maintained under a specific light intensity, the effect of light wavelength on the mycelial growth was examined. The results were also recorded in Table 2. As shown in Fig. 4a, when the light intensity was 20 μmol photon m^−2^ s^−1^, although all light groups resulted in a slightly smaller colony diameter than the control group, only the green light slowed down the mycelial growth significantly, by a 4.4% decrease (*P* = 0.0153). When the intensity increased to 40 μmol photon m^−2^ s^−1^, the green light and the blue light inhibited the mycelial growth (*P* = 0.0216 and 0.0398, respectively), while the colony diameter of the red light group was very nearly the same as that of the control group. After the irradiation at a light intensity of 60 μmol photon m^−2^ s^−1^, the mycelial growth of the red light group remained similar to that of the darkness group, while the green light group and the blue light group kept their slower growth. At the light intensity of 80 μmol photon m^−2^ s^−1^, only the blue light (80 μmol photon m^−2^ s^−1^) suppressed the mycelial growth, so growth of the blue light group was significantly different from those of the other two light groups. As shown by the four panels in Fig. 4, no consistent trend was found between the different light wavelength groups. This indicated that within the setting of light intensity, the mycelial growth of A. oryzae GDMCC 3.31 was influenced by different light wavelengths.

### Effect of light irradiation on conidium production.

In addition to mycelial growth, reproduction is an important life stage of fungi. A. oryzae reproduces asexually in the form of conidia. Specifically, a part of the mycelium is specialized into aerial hyphae. The top of aerial hyphae further forms the conidiophore stem. Then, the yellow-green conidiophore will live on the top of the conidiophore stem, spread with the wind easily, and will germinate into a new generation of mycelium in a suitable environment in the future. The effect of light irradiation on the conidium production of A. oryzae GDMCC 3.31 is discussed below.

### Effects of different light intensities on conidium production under the same light wavelength.

**FIG 5:**
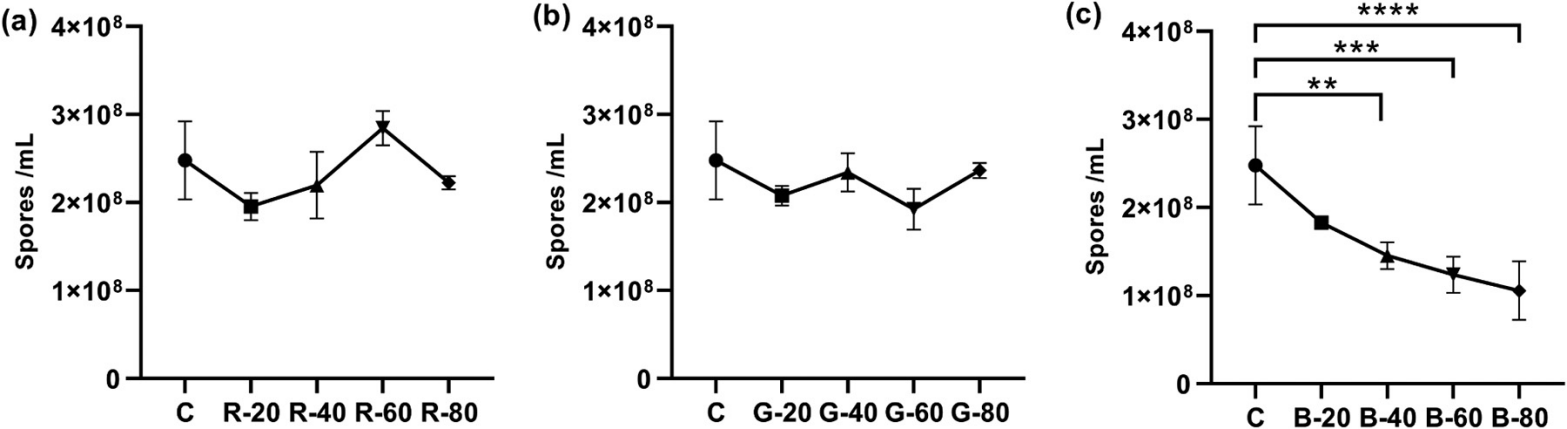
Number of conidia produced in each group under different light intensities. (a) Number of conidia produced in the red light groups at different light intensities. (b) Number of conidia produced in green light groups. (c) Number of conidia produced in blue light groups. The points on the *x* axis from left to right represent the dark control group and the light groups at intensities of 20, 40, 60, and 80 μmol photon m^−2^ s^−1^. The error bars represent the standard deviation. Significance: **, *P* < 0.01; ***, *P* < 0.001; ****, *P* < 0.0001.

Results of the light intensity experiments were recorded in Table 3. As shown in Fig. 5a, although the conidia produced in the groups under red light fluctuated relatively with the increased intensities, intensity had no significant difference compared with the dark group. The conidia harvested from the green light groups did not change much with increased light intensity. Except for the green light group (60 μmol photon m^−2^ s^−1^), the average number of conidia in green groups was greater than 2 × 10^8^ spores/ml. However, the blue light group result was different from those of the other two light groups. Despite the fact that the blue light group (20 μmol photon m^−2^ s^−1^) had no significant difference, blue light resulted in fewer conidia than the control group, as shown in Fig. 5c. There was a consistent decrease in the number of conidia with increased blue light intensity from 20 to 80 μmol photon m^−2^ s^−1^ compared to the darkness group. Especially, the count of the conidia of the blue light group (highest intensity of 80 μmol photon m^−2^ s^−1^) was 57.4% less than that in the cultures maintained under darkness (*P* < 0.0001). These results implied that cultivation under a certain intensity of blue light could adversely affect the final conidium production of A. oryzae GDMCC 3.31, and the degrees of response of A. oryzae GDMCC 3.31 to various blue light intensities were different, while the red light and the green light had no such obvious influence.

**TABLE 3:** Conidia under different light wavelengths and light intensities

Light intensity (μmol photon m^−2^ s^−1^)	No. of spores/ml under^*a*^:			
	Darkness	Red light	Green light	Blue light
20	2.48 × 10^8^ ± 4.4 × 10^7^ ^ABC^	1.95 × 10^8^ ± 1.6 × 10^7^	2.08 × 10^8^ ± 1.1 × 10^7^	1.83 × 10^8^ ± 5.9 × 10^6^
40		2.20 × 10^8^ ± 3.8 × 10^7^ ^D^	2.34 × 10^8^ ± 2.2 × 10^7^ ^E^	1.45 × 10^8^ ± 1.5 × 10^7^ ^ADE^
60		2.84 × 10^8^ ± 2.0 × 10^7^ ^FG^	1.92 × 10^8^ ± 2.3 × 10^7^ ^G^	1.24 × 10^8^ ± 2.1 × 10^7^ ^BF^
80		2.22 × 10^8^ ± 7.5 × 10^6^ ^H^	2.36 × 10^8^ ± 8.5 × 10^6^ ^I^	1.06 × 10^8^ ± 3.3 × 10^7^ ^CHI^

a The values are expressed as means ± standard deviations. Each treatment was repeated at least 3 times. Different letters in columns and rows represent statistically different mean values (*P* < 0.05).

### Effects of different light wavelengths on conidium production under the same light intensity.

**FIG 6:**
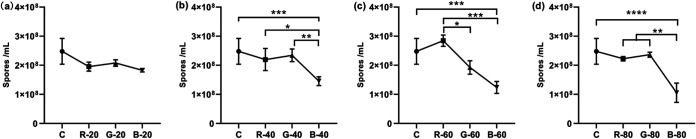
Numbers of conidia produced in groups under different light wavelengths. Panel a shows the numbers of conidia of the groups under different light wavelengths at the same intensity (20 μmol photon m^−2^s^−1^). Panels b, c, and d are marked in the same way at respective intensities of 40, 60, and 80 μmol photon m^−2^ s^−1^. The error bars represent the standard deviation. Significance: *, *P* < 0.05; **, *P* < 0.01; ***, *P* < 0.001; ****, *P* < 0.0001.

When the light conditions were maintained at low intensity, the effect of light wavelength on the conidium production was examined. As shown in Fig. 6a and Table 3, after strains were irradiated at low light intensity (20 μmol photon m^−2^ s^−1^), conidia harvested from those light wavelength groups were a little different. When the intensity increased to the middle level (40 μmol photon m^−2^ s^−1^), blue light showed an influence different from those of other light wavelengths. The blue light group had significant suppression of conidium production, compared not only to the darkness group but also to the other light groups. In addition, this trend was maintained as the light intensity continued to increase. Red light (40, 60, and 80 μmol photon m^−2^ s^−1^) showed no effect on conidium production, while production was statistically different from those of the other light groups in Fig. 5b to d. Those results demonstrated that the conidium production of A. oryzae GDMCC 3.31 could be affected by different light wavelengths. Besides, this effect from different light wavelengths may have a light intensity threshold.

### Interaction contribution between light wavelength and intensity.

From the above results, it was not difficult to find that both light wavelength and intensity have an impact on mycelial growth and conidium production. All of those results, such as blue light irradiation (80 μmol photon m^−2^ s^−1^) resulting in the lowest production of conidia, may be because of the combined contribution of light’s wavelength and intensity. Therefore, two-way ANOVA was performed, respectively, on colony diameter and the number of conidia to analyze the effect of the interaction between wavelength and intensity.

### Interaction effect of light wavelength and intensity on mycelial growth.

As recorded in Table 4, a significant influence on the mycelial growth of A. oryzae GDMCC 3.31 existed from the interaction between light wavelength and intensity (*P* = 0.0003). This suggested that when studying the light response of A. oryzae, light wavelength and intensity should be considered two independent and interactive factors. If one aspect of A. oryzae was investigated at multiple light wavelengths, it is necessary to make sure that the light intensity is consistent.

**TABLE 4:** Two-way ANOVA results for colony diameter

Source of variation	% of total variation	*P* value^*a*^	Significant?
Interaction	24.38	0.0003***	Yes
Light intensity	19.08	0.0001***	Yes
Light wavelength	17.70	<0.0001****	Yes

a Significance: ***, *P* < 0.001; ****, *P* < 0.0001.

**FIG 7:**
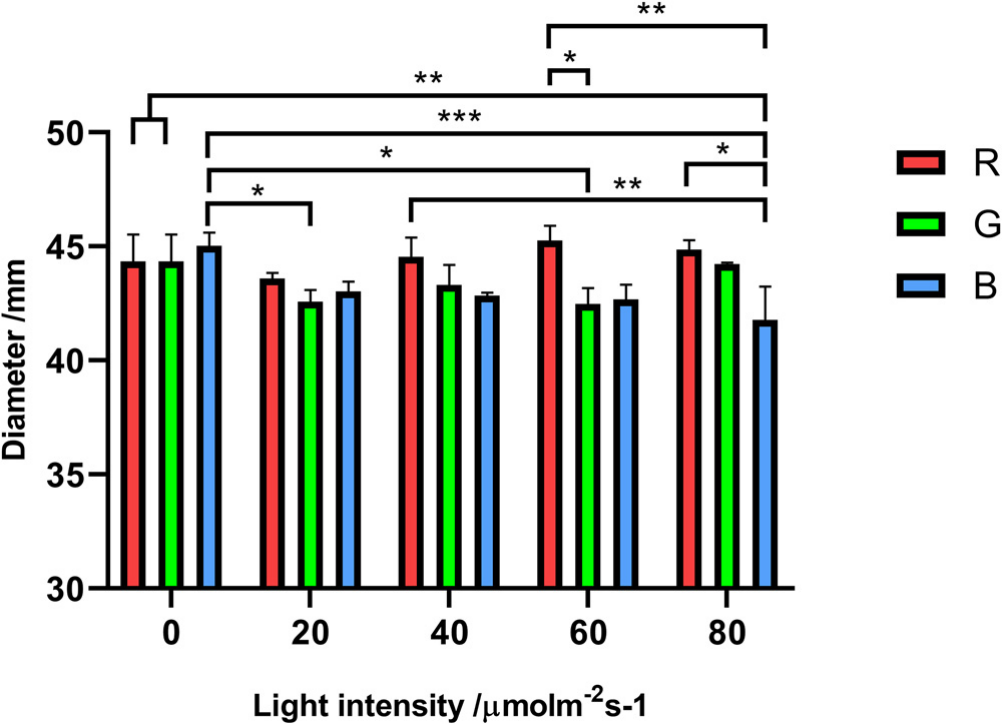
Two-way ANOVA of all colony diameter results. The 0 on the *x* axis represents the darkness control group. The *y* axis represents the average diameter of the colony (mm). The R, G, and B bars, respectively, represent the red, green, and blue light groups. The error bars represent the standard deviation. Significance: *, *P* < 0.05; **, *P* < 0.01; ***, *P* < 0.001.

Tukey’s multiple comparisons were performed, as shown in Fig. 7. The red light still had no significant difference with the control group. The inhibition by green light (wavelength of 520 nm and intensities of 20 and 60 μmol photon m^−2^ s^−1^) and blue light (wavelength of 475 nm and intensity of 80 μmol photon m^−2^ s^−1^) of mycelial growth was maintained as well. In consideration of the effect of a two-factor interaction, some results were different from the results of the one-way ANOVA. No significant difference was found between the green light group (40 μmol photon m^−2^ s^−1^) and the control group or the blue light group (60 μmol photon m^−2^ s^−1^) and the control group, which were different from the results in the sections “Effects of different light intensities on mycelial growth under the same light wavelength” and “Effects of different light wavelengths on mycelial growth under the same light intensity.”

### Interaction effect of light wavelength and intensity on conidium production.

**FIG 8:**
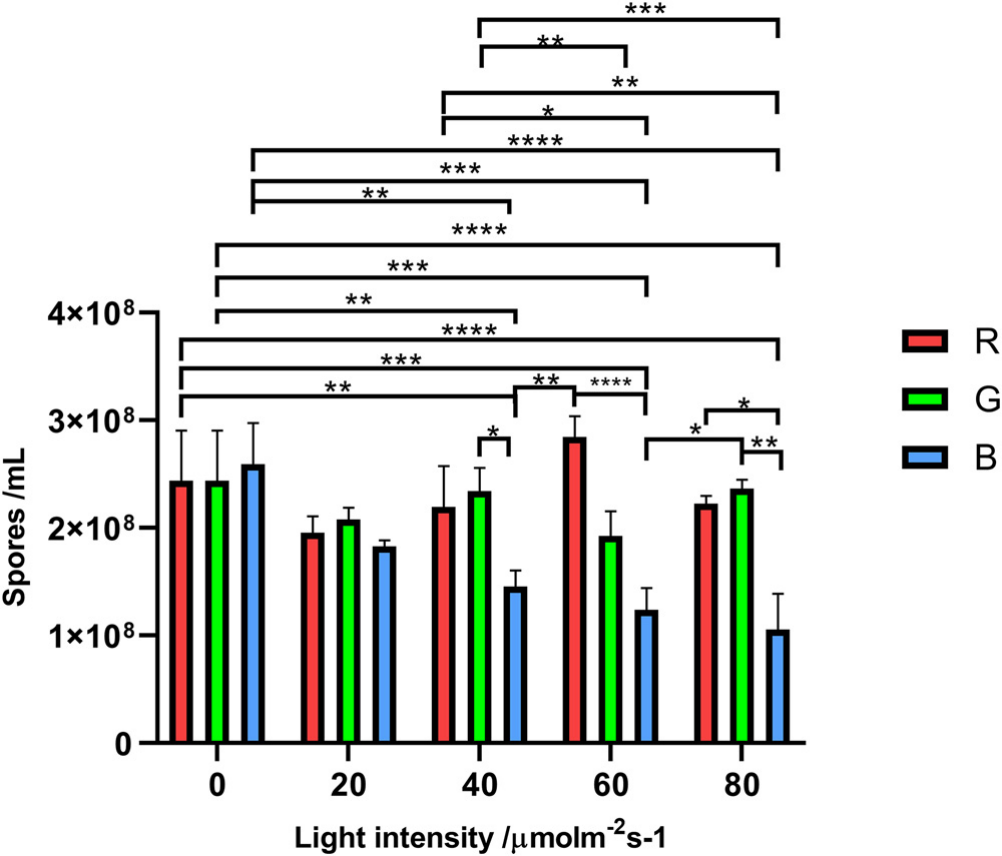
Two-way ANOVA of all conidial number results. The 0 on the *x* axis represents the darkness control group. The *y* axis represents the average diameter of the colony (mm). The R, G, and B bars, respectively, represent the red, green, and blue light groups. The error bars represent the standard deviation. Significance: *, *P* < 0.05; **, *P* < 0.01; ***, *P* < 0.001; ****, *P* < 0.0001.

As shown in Table 5 and Fig. 8, an obvious interaction effect from these two factors to the number of conidia was observed (*P* < 0.0001). This also implied that light wavelength and intensity should be considered comprehensively in terms of the conidium production of A. oryzae GDMCC 3.31. After Tukey’s multiple comparisons, the red light and the green light still had no significant difference compared, respectively, to the darkness. There was obvious suppression by the blue light of conidium production compared to the darkness, which was consistent with the results of the one-way ANOVA discussed above.

**TABLE 5:** Two-way ANOVA results for conidium number

Source of variation	% of total variation	*P* value^*a*^	Significant?
Interaction	25.58	<0.0001****	Yes
Light intensity	22.05	<0.0001****	Yes
Light wavelength	22.06	<0.0001****	Yes

a Significance: ****, *P* < 0.0001.

## DISCUSSION

The effects of light on fungal biology are the result of coordinated transcriptional regulation and activation of signal transduction pathways. Therefore, exploring the photoresponse of Aspergillus oryzae is an important step in studying its photobiological effects and lays the foundation for determining its photoreceptors later. At the same time, this kind of study may bring some inspiration to the optimization of related technologies in the food brewing industry. Our results showed that blue light at a certain intensity inhibits the mycelium growth and conidium production of A. oryzae GDMCC 3.31, while GDMCC 3.31 is insensitive to red light.

Based on the monochromatic light sources, our results could bring some thoughts on the previous studies, which used white light sources as research means. The repression in our study is similar to the light response of A. oryzae RIB40 and F6, which produced fewer conidia under a continued white fluorescent light irradiation (6, 17, 19); this may because white light includes the spectrum range of blue light. Besides, the light response of A. oryzae GDMCC 3.31 was not the same as that of the other A. oryzae strains. For example, A. oryzae RIB40 produced more conidia under darkness than under red light, while the number of conidia produced by A. oryzae GDMCC 3.31 showed no significant difference. The reason may be from a difference in the strain itself, and some researchers also found a similar phenomenon (20, 24). Whether there are intraspecies differences in light response, further research is needed to confirm this.

It is still difficult to explain why the mycelial growth and sporulation of A. oryzae GDMCC 3.31 respond differently to red light and blue light, due to their being less study of the molecular mechanism and photoreceptor involved. However, we could make some assumptions based on the results of the research on the light response of Aspergillus. Generally, fungi contain at least two types of photoreceptors, which are characterized by containing chromophores, such as the blue light receptors with flavin, the green light sensor (also called opsin) with the retina, and the red light receptors (photochrome) with linear tetrapyrrole. Currently, the mainstream explanation of the light response is that light irradiation causes a change of the chromophore, which in turn leads to primary conformational changes of the photoreceptor. Signals are then followed by different output mechanisms and ultimately affect gene regulation. In Aspergillus nidulans, the phytochrome FphA perceives the red light, and then the high osmotic pressure glycerol (HOG) mitogen-activated protein (MAP) kinase pathway transmits signals to the nucleus, where AtfA transcription factors activate light-induced genes to balance asexual development and sexual development (1, 25). Since red light (630 nm) did not affect the sporulation of A. oryzae GDMCC 3.31, and blue light (475 nm) inhibited conidium production, it is suspected that A. oryzae GDMCC 3.31 has a blue light receptor instead of the red light receptor. Verification of this hypothesis is our next study. If confirmed, it could be further inferred that when the blue light receptor senses blue light irradiation, the signal is transmitted to the central regulatory module related to spores (such as the BrlA→AbaA→WetA regulatory cascade) (13, 15) through a certain pathway, which downregulated the related genes and ultimately led to a significant reduction in the number of conidia produced.

Studies have shown that A. oryzae is a morphological variant of Aspergillus flavus (26, 27). The fungi both belong to the yellow-green group of Aspergillus and are similar in morphology and genome, since it has been demonstrated that production by A. flavus of secondary metabolites, such as aflatoxin, was light responsive. Blue-green light irradiation at low intensity (4 μmol photon m^−2^ s^−1^) resulted in the highest level of aflatoxin synthesis in A. flavus (6). Illumination with two LED lights (50 W, 2,250 lx; Stella) reduced A. flavus mycelial biomass yield while promoting conidiation and aflatoxin production (9). Therefore, the secondary metabolites of Aspergillus oryzae may also respond to light irradiation, which arouses our interest. In the soy sauce industry, koji is one of the important stages of production. High-quality koji has abundant enzymes, such as protease, amylase, pectinase, and cellulase, and the activities of those enzymes are high. These enzymes and their products play a key role in forming the flavor quality of soy sauce (28, 29). In recent years, some researchers have used mixed strains for fermentation to improve the enzyme variety and activity (30–32). The koji obtained by mixed fermentation of A. oryzae and Aspergillus niger was confirmed to produced more total phenol, more total flavonoid, and higher antioxidant activity than the single-strain fermentation (31). Besides, improvement of acid protease production was found in the mixed culture of A. niger and A. oryzae using solid-state fermentation (32). Studies have also shown that A. niger has a positive photogenic response to white light (33), and the production of conidia and glucoamylase was promoted by continuous illumination with blue light (34). Considering blue light inhibited the mycelial growth and conidiation of A. oryzae GDMCC 3.31, blue light can be used to regulate the koji of a mixed strain of A. oryzae and A. niger. For example, by introducing blue light irradiation at a certain time, the dominant strain in the koji would be changed, thus secreting more of the expected enzymes and finally improving the quality of the koji.

In subsequent work, we will focus on several aspects, including the influence of different light conditions on A. oryzae GDMCC 3.31 metabolism and comparison with other common strains. We will also try to do some exploration of gene expression and find related photoreceptors.

### Conclusion.

In conclusion, those results demonstrated that the mycelial growth of the A. oryzae GDMCC 3.31 was inhibited by green light (wavelength of 520 nm and intensities of 20 and 60 μmol photon m^−2^ s^−1^) and blue light (wavelength of 475 nm and intensity of 80 μmol photon m^−2^ s^−1^). Conidium production was suppressed by the blue light (wavelength of 475 nm and intensities of 40, 60, and 80 μmol photon m^−2^ s^−1^). Furthermore, with increasing intensity of blue light irradiation, the inhibition of conidium production increased. When the strain was irradiated with blue light (80 μmol photon m^−2^ s^−1^), the number of conidia produced was 57.4% less than that of the darkness group. However, within our set range of intensity, the strain was insensitive to red light (630 nm) in both mycelium growth and conidium production. Besides, further two-way ANOVA showed that light wavelength and intensity have an interaction effect, not only on mycelium growth but also on conidium production.

## MATERIALS AND METHODS

### Fungal species.

Aspergillus oryzae GDMCC 3.31 (also designated CGMCC 3.951) was obtained from the China Guangdong Microbial Culture Collection Center: the strain is commonly used in the Chinese food brewing industry. Each sample was incubated on a peptone-dextrose agar (PDA) (CM123; Beijing Land Bridge Company, China) slant at 28°C for 5 days. After two subcultures, the third generation of A. oryzae was obtained and prepared for the spore suspension by using a sterile water solution containing 0.002% (vol/vol) Tween 80 and 0.5% (wt/vol) NaCl. Spores were stored at 4°C.

### Culture conditions.

PDA plates without any additional reagents were prepared in advance. The spore suspension was diluted to a concentration of 1 × 10^7^ CFU/ml as a conidial inoculum. Each plate was inoculated with 10 μl of inoculum solution and transferred to incubators for culturing until 72 h. Conditions of specific irradiation (a certain wavelength and intensity) and 30°C were set during the cultivation process.

To studied the effect of different light conditions on mycelial growth, the colony diameter was used as the evaluation. After the incubation, the colony diameter in each plate was measured using a vernier caliper by randomly choosing 4 directions to measure, and then the average value was calculated and marked as the colony diameter of the sample. Three parallel samples were finally averaged to result in the experiment value.

To test the influence of different light conditions on the germination of conidia, the number of conidia produced by point incubation with and without light irradiation was investigated. After 72 h of incubation, the conidia grown on each plate were washed with 5 ml of sterile water containing Tween 80. Then the solution was filtered to obtained spore suspensions. It was then properly diluted by a certain multiple and counted with a hemocytometer to obtain the number of conidia, which was recorded in Table 3.

### Light conditions and apparatus.

The light conditions were provided by light incubators (Spectracell-MU250L; LightEngin Technology, China). The LED light sources are on the bottom of the incubator and can be replaced (Fig. 1). Three wavelengths and four intensities were combined, respectively, to form 12 different light conditions. In detail, the three central wavelengths of the LEDs were 475, 520, and 630 nm, and the four intensities were 20, 40, 60, and 80 μmol photon m^−2^ s^−1^. Each culture plate, except under the dark condition, was continuously irradiated with the specific light condition during the culture. All light intensities were fine-tuned with a photosynthetic active radiation detector (PQS1; BLH Technology Company). The irradiance of 20, 40, 60, and 80 μmol photon m^−2^ s^−1^ was measured with a spectral irradiance colorimeter (SPIC-300; Everfine Photo-E-Info Co., Ltd.).

### Statistical analyses.

The darkness group was set as the control group in all comparisons. One-way ANOVA was used to analyze the difference between the groups when the light wavelength or light intensity was maintained the same. To further analyze the interaction effect between light wavelength and intensity, a two-way ANOVA was performed. All experiments were repeated at least in triplicate, and each condition group was prepared in three parallel samples. Results are presented as means ± standard deviations. The symbols *, **, ***, and **** represent *P* values of <0.05, <0.01, <0.001, and <0.0001, respectively.
